# Neuromuscular fatigue reduces force responsiveness when controlling leg external forces

**DOI:** 10.14814/phy2.70498

**Published:** 2025-08-22

**Authors:** Pawel Kudzia, James M. Wakeling, Stephen N. Robinovitch, J. Maxwell Donelan

**Affiliations:** ^1^ Department of Engineering Science Simon Fraser University Burnaby British Columbia Canada; ^2^ Department of Biomedical Physiology and Kinesiology Simon Fraser University Burnaby British Columbia Canada

**Keywords:** force modulation, ground reaction forces, injury prevention, motor control, neuromuscular fatigue, responsiveness

## Abstract

Agile movement requires precise control of ground reaction forces, yet the impact of neuromuscular fatigue on this control remains incompletely understood. We investigated how leg muscle fatigue affects the nervous system's ability to modulate vertical ground reaction forces, hypothesizing that fatigue would impair responsiveness, and accuracy. Eighteen healthy participants (11 W, 7 M; age: 26 ± 3.8 yr) performed force‐matching tasks using a custom apparatus that constrained body movement while allowing isolated leg force production. Participants force matched visual step targets before and after fatigue, which we induced by sustained submaximal contractions. We then quantified responsiveness (rise time, bandwidth) and accuracy (overshoot, steady‐state error, variability). Fatigue reduced MVC force by 26.1 ± 12.3% (from 2.95 ± 0.58 to 2.20 ± 0.47 body weights; *p* = 4.70e‐6) and impaired responsiveness, with rise time increasing 23.3 ± 41.0% (from 303 ± 100 to 373 ± 94 ms; *p* = 6.00e‐4) and bandwidth decreasing 25.2 ± 17.5% (from 1.33 ± 0.60 to 1.00 ± 0.25 Hz; *p* = 4.83e‐2). Notably, accuracy remained largely unchanged, with only a transient decrease in overshoot from 4.30 ± 2.62% to 2.56 ± 1.19% (*p* = 0.047). These findings show neuromuscular fatigue selectively impairs the speed of leg force adjustments while preserving accuracy, possibly through compensatory strategies. This dissociation has implications for understanding motor control deficits and injury risk during fatigue.

## 1 INTRODUCTION

Agility in many animals, including humans, depends on the precise control of external leg forces. While we can push against the ground with any part of our bodies, we primarily use our legs to generate force through our feet. The resulting ground reaction force, a vector quantity, accelerates the body, and influences movement. Rapid and accurate control of this force is essential for agility (Kudzia et al., 2022). Examples include accelerating from a standstill, jumping over obstacles, and quickly changing direction—all of which require precise regulation of external leg forces. However, while we know fatigue reduces maximum force capacity, we lack an understanding of how it affects the nervous system's ability to rapidly and accurately modulate forces at the submaximal force levels where, most athletic movements occur.

Neuromuscular fatigue disrupts force control through multiple physiological mechanisms. While fatigue's impact on maximum capacities is well‐established, reducing peak force generation (Nocella et al., 2011), slowing maximum shortening velocities (Boccia et al., 2017; Jones, 2010) and decreasing maximum power output (Nocella et al., 2011), its effects on submaximal force control remain poorly understood. During athletic movements, fatigue disrupts sensorimotor control through several pathways: slowed muscle reaction times (Benesch et al., 2000), altered proprioception (Mohammadi & Roozdar, 2010), and delayed neuromuscular responses (Wilkins et al., 2004). These changes manifest in real‐world performance decrements, such as soccer players' reduced balance control when fatigued (Zemková & Hamar, 2009) and basketball players' altered landing mechanics late in games (Benesch et al., 2000). However, a fundamental question remains: does fatigue impair our ability to target the correct force levels (accuracy), our ability to rapidly achieve those forces (responsiveness), or both?

Fatigue impairs force control by disrupting both feedforward neural drive and feedback sensory regulation. Neural drive, which governs signal strength from the brain to muscles, decreases with fatigue due to reduced neurotransmitter release, altered motor neuron firing patterns, and diminished muscle fiber sensitivity (Taylor et al., 2016). This leads to weaker and less coordinated force production (Bigland‐Ritchie, 1984; Ranieri & Di Lazzaro, 2012). Simultaneously, fatigue weakens sensory feedback gains that are essential for adjusting muscle force based on sensory input, such as muscle length or velocity (Azim & Seki, 2019). As these gains decline, the ability to make rapid and accurate force corrections diminishes, resulting in slower responses and greater force variability (Franklin, 2008). To compensate, individuals may alter movement patterns or rely more on alternative muscle groups, often sacrificing efficiency and precision.

The impact of fatigue on fine force control remains insufficiently explored, particularly for submaximal force regulation over time. Previous studies show fatigue increases force variability during sustained isometric contractions and alters motor unit firing behavior (Adam & De Luca, 2005; Contessa et al., 2009). However, most research focuses on sustained contractions rather than the ability to precisely regulate submaximal forces, which are essential for maintaining stability and performing functional tasks. While fatigue links to deficits in proprioception and balance, less is known about how it affects the nervous system's ability to adjust external forces with accuracy and responsiveness (Johnston 3rd et al., 1998). Understanding these effects is critical, as impaired force control under fatigue could contribute to movement instability, decreased performance, and increased injury risk.

Our study aimed to characterize fatigue's effect on the nervous system's ability to responsively and accurately control vertical external leg forces. We hypothesized that neuromuscular fatigue would impair both the responsiveness (speed of force adjustments) and accuracy (precision and consistency) of submaximal leg force control. Specifically, we predicted that fatigue would lead to slower rise times, reduced bandwidth, increased force variability, and larger steady‐state errors when matching target forces.

## 2 METHODS

### 2.1 Participants

We recruited 18 healthy participants for the study (11 women, 7 men). For the male participants (all men), we found an age of 27.9 ± 4.6 years, body mass of 79.0 ± 8.9 kg, height of 180.4 ± 11.9 cm, and shoe size of 10.5 ± 1.0 US sizing (mean ± std). For the female participants (all women), we found an age of 25.6 ± 3.2 years, body mass of 71.0 ± 19.6 kg, height of 166.4 ± 9.7 cm, and shoe size of 7.7 ± 1.6 US sizing (mean ± std). The average of the combined group showed an age of 26.5 ± 3.8 years, body mass of 74 ± 16 kg, height of 171 ± 12 cm, and shoe size of 9 ± 2 US sizing (mean ± std). Participants provided verbal and written informed consent before participating.

We established inclusion criteria as: (1) age 18‐40 years, (2) ability to perform single‐leg stance for at least 60 seconds, (3) absence of current pain or discomfort in the lower extremities or back, and (4) ability to understand and follow experimental instructions. We applied exclusion criteria including: (1) history of lower extremity surgery, (2) musculoskeletal injury to the lower extremities or back within the past 6 months that required medical attention or time off from normal activities, (3) diagnosed neurological conditions affecting balance or motor control, (4) current use of medications known to affect neuromuscular function, and (5) pregnancy. Participants represented a range of activity levels from recreationally active (engaging in physical activity 2‐3 times per week) to highly active (daily physical activity). However, we did not formally assess or control for training status. All participants reported their ability to perform activities of daily living without limitation.

### 2.2 Experimental design

**FIGURE 1:**
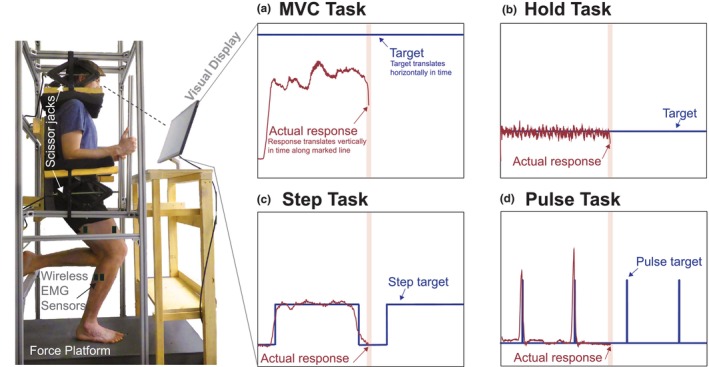
Experimental apparatus for measuring leg force control. Participants stood on their right leg on a force platform while constrained at the shoulders and forearms by adjustable scissor jacks mounted to an aluminum frame. Real‐time visual feedback displayed the applied vertical force and target force. Four task types were performed: (a) MVC Task: Maximal push for 10 s toward unattainable target (5.0 Bodyweight (BW)); (b) Hold Task: Maintain 1.4 BW for 73 s; (c) Step Task: Match 10 step targets requiring both speed and accuracy; (d) Pulse Task: Match 10 brief pulse targets requiring rapid response.

To characterize leg force control, we tested the step response of participants as they selectively controlled external leg forces. We used a custom apparatus (Figure 1) that we had previously built (Kudzia, 2023; Kudzia et al., 2022). The apparatus consisted of a ground‐embedded force plate (Bertec Corporation, Ohio, USA) that participants stood on, constrained in both the vertical and horizontal directions. We connected the force plate to a computer through a data acquisition unit (USB‐6229, National Instruments Corporation, Texas, USA), which sampled data at 1000 Hz. Participants were able to exert a variable but controlled external force vector onto the ground by selectively pushing down with their leg. To support force generation and ensure stability, participants were allowed to grip the apparatus and use their shoulders for additional support during all tasks, as shown in Figure 1. This design helped participants generate higher and more stable vertical forces, particularly during maximal effort trials, while maintaining safety and consistency across participants. The apparatus featured rigid supports that constrained participants at the shoulders and forearms, preventing vertical and horizontal movement of their upper body. Although control of force‐position in the medial‐lateral and anterior–posterior directions (i.e., center of pressure control) and control of all three orthogonal force magnitude components of the external force vector are necessary, our work here focused only on controlling vertical force magnitude. In our prior work, we characterized the control of different step sizes of medial–lateral and anterior–posterior force positions and the control of a range of submaximal vertical force magnitudes. We found negligible differences in control characteristics for these different components of the external force vector (Kudzia et al., 2022). By focusing on only the vertical component of force, our findings will represent the range of control characteristics for all aspects of controlling the external force vector at submaximal forces and any control changes resulting from fatigue.

We recorded surface EMG signals from six muscles of the right leg using wireless EMG sensors (Trigno, Delsys Inc., Natick, MA, USA). Each sensor contained four silver bar electrodes (10 mm inter‐electrode distance) that we aligned perpendicular to the muscle fiber direction to optimize signal quality (Delsys Trigno Wireless EMG Sensors, n.d.). We placed sensors on the muscle belly of vastus medialis, vastus lateralis, rectus femoris, biceps femoris, gastrocnemius medialis, and gastrocnemius lateralis, identifying optimal placement through palpation while avoiding tendons and muscle edges (Hermens et al., 2000). We prepared the skin by removing excess hair and cleaning it with isopropyl alcohol to ensure good electrode‐skin contact. We attached the sensors using double‐sided adhesive interfaces (Delsys Inc.), which provided stable mechanical coupling without requiring conductive gel. We sampled EMG signals at 2000 Hz and synchronized them with force plate data using a hardware trigger implemented in MATLAB (2022b). The wireless system introduced a 500 μs inter‐sensor transmission delay, which we accounted for during post‐processing to ensure accurate temporal alignment of muscle activation patterns.

We then fitted participants into the experimental apparatus. When fitting participants, we adjusted the apparatus such that participants maintained an approximately 15‐degree knee flexion angle in their right leg, a posture similar to that assumed by a runner at the start of the stance phase (Fukuchi et al., 2017; Kudzia et al., 2022). We standardized this measure by using a goniometer and adjusting their position as needed. In this fitting process, we moved components of the apparatus to push down on the participant's shoulders and up on their arms by manually rotating four scissor jacks (Figure 1). Our goal was to ensure that we fixed participants tightly within the apparatus and were unable to move side‐to‐side or vertically.

To assess the effects of leg fatigue on the control of leg external force, we commanded four types of force control tasks. We had participants perform the experiments by standing on their right leg in all tasks. We used visual feedback to allow participants to compare their actual vertical force magnitude to a target we instructed them to match. We provided real‐time visual feedback using a monitor, where our custom‐developed software displayed both the real‐time feedback of the vertical force magnitude signal that the participant's whole foot exerted onto the force plate and the target that the participant tried to rapidly and accurately match. For visual display, we normalized the real‐time signal to each participant's body weight and filtered the raw force signal using a low‐pass fourth‐order Butterworth filter with a cut‐off frequency of 10 Hz. We programmed the real‐time signal to display at the center of the screen and constrained it only to move up and down as the participant pushed more or less against the force plate. We programmed the target to slide past the real‐time signal, giving participants time to view any upcoming changes in the target before they occurred.

In the first force control task, the Maximum Voluntary Contraction Task (MVC Task), we evaluated the maximum effort of voluntary force that the leg could exert on the ground. To collect the MVC Task, we asked participants to push down against the ground as hard as possible and try to reach an unattainable target of 5 times their body weight (5.0 BW) displayed on the screen. From pilot experiments, we found that providing participants with a target to reach, even if it was not attainable, motivated them to push harder to try and reach it. Each MVC Task lasted 10 s as participants pushed down with their whole foot against the ground as hard as possible. We asked participants to increase their force in the first ~3 s from zero to maximum and then sustain this hold for 7 s.

We designed the second force control task, the Hold Task, to fatigue each participant's right leg. In the Hold Task, we asked participants to match and hold a target equal to their body weight plus 25% of their Pre‐Fatigue maximum voluntary contraction, as determined in the MVC Task before the onset of any leg fatigue (Taylor & Gandevia, 2008). This type of sustained voluntary contraction progressively recruits additional motor units as initially active units fatigue, leading to muscle fatigue in the active muscles (Enoka & Duchateau, 2008; Taylor & Gandevia, 2008). This method of fatigue was effective in a similar study (Salomoni & Graven‐Nielsen, 2012). For each Hold Task, we asked participants to hold their force level to the best of their ability to match the target for a total duration of 73 s, the same duration as the third leg force control task. If the participant was not able to hold the force dropping below 10% of the target, we provided verbal encouragement until the 73 s were finished. All participants were able to reach the end of this timeframe.

In the third leg force control task, the Step Task, we probed force control characteristics by commanding target step changes in leg vertical force magnitude. Because both initial speed to the new target and steady‐state accuracy around the new target are objectives, we anticipate that the nervous system will depend on feedback control to accomplish this task. In a single Step Task, the target step function consisted of a square wave of 10 matching‐size target steps, each 4 s in duration, with 3 s between steps (totalling 73 s per task). The lower value of the step target was 1.0 BW, and the upper value (i.e., the size of the target) was 1.4 BW. We selected the target step size based on a previous experiment where we studied step responses by commanding a range of target step sizes, both smaller and larger than the magnitude chosen here (Kudzia et al., 2022). We observed minor changes in control characteristics between targets of varying step sizes, so we chose an intermediate step size for this experiment.

In the fourth force control task, which we referred to as the Pulse Task, we aimed to probe rapid force control by asking participants to respond as rapidly as possible to changes in the target. Because this task emphasizes the initial speed of response over steady‐state accuracy, we anticipate that the nervous system will emphasize feedforward control over feedback control when accomplishing this task (Kuo, 2002). The target in this task resembled a pulse, changing momentarily for 0.05 s from 1.0 BW to the target step size of 1.4 BW (the same step size as in the Step Task). In a single Pulse Task, we programmed the target pulse to go from body weight to the target step size 10 times over 73 s.

### 2.3 Experimental protocol

**FIGURE 2:**
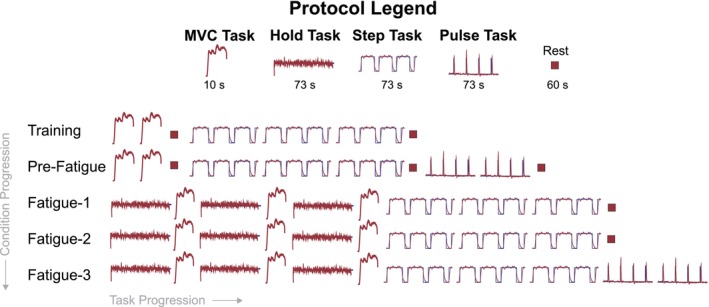
Experimental protocol showing five conditions: Training, Pre‐Fatigue, Fatigue‐1, Fatigue‐2, and Fatigue‐3. Each condition consisted of combinations of four tasks: MVC Task, Hold Task, Step Task, and Pulse Task. Within‐condition progression flows left to right; between‐condition progression flows top to bottom.

We conducted the experiment in a single session. The session included five conditions: Training, Pre‐Fatigue, Fatigue‐1, Fatigue‐2, and Fatigue‐3 (Figure 2). We started the study with the Training condition to familiarize participants with the experiment. In this condition, we explained and demonstrated how the visual feedback and force plate pushing worked. Then, we asked each participant to perform two repetitions of the MVC Task followed by three repetitions of the Step Task. Our previous work suggests that this is enough training in the apparatus to minimize any learning effects (Kudzia et al., 2022). After the Training condition, we gave participants a 1‐minute break to stand freely on both feet in the apparatus. Next, in the Pre‐Fatigue condition, we had participants perform two repetitions of the MVC Task followed by a 1‐minute break, then three Step Tasks followed by a 1‐minute break, and then two Pulse Tasks. We then gave participants a 1‐minute break to rest before the next condition. Next, we started the sequence of three fatigue conditions (Fatigue‐1, Fatigue‐2, Fatigue‐3). Each Fatigue condition consisted of a Hold Task followed by an MVC Task. We repeated this three times within each Fatigue condition without rest (see Figure 2 for full details of the study design). After the third and final MVC Task in each Fatigue condition, we immediately had each participant perform three repetitions of the Step Task to probe force control. For each Fatigue condition, we repeated this same sequence of tasks. On the third Fatigue condition and after the final Step Task, participants performed two Pulse Tasks. We gave participants a 1‐minute break between Fatigue conditions.

## 3 DATA ANALYSIS

**FIGURE 3:**
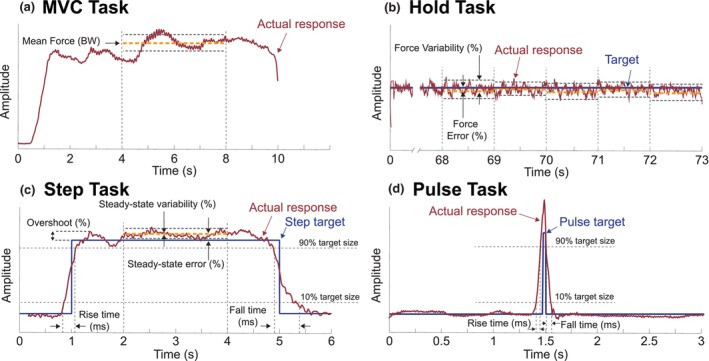
Analysis windows and measured parameters for the four leg force control tasks. (a) MVC Task: Mean force calculated between 4 and 8 s. (b) Hold Task: Force variability calculated for each 1‐second interval after the initial 5 s. (c) Step Task: Responsiveness (rise time, fall time, bandwidth) and accuracy (overshoot, steady‐state error, steady‐state variability) measured for each step response. (d) Pulse Task: Responsiveness parameters measured for each pulse target.

For each MVC Task, we evaluated the mean vertical force output as one objective measure of fatigue (Figure 3a). We quantified the mean vertical force output by taking the signal's average during the MVC Task's midway point between the 4 and 8 s time interval. We considered a minimum of 5% drop (from the previous trial) as a relevant drop in MVC, suggesting local fatigue.

For each Hold Task, we evaluated the force variability of the response as another objective measure of fatigue (Figure 3b). Increases in force variability during isometric holds are associated with increased muscle fatigue (Contessa et al., 2009). We segmented the data from each Hold Task into 1‐second segments to quantify changes over time. We omitted any further analysis from the first 5 s of this signal as participants adjusted their vertical force to match the target during this time frame. For each 1‐second segment, we then quantified force variability by calculating the standard deviation of the signal during each 1‐second segment, dividing this by the mean of the signal during that time frame, and multiplying this value by 100. This expresses variability as a percentage of the force magnitude.

For each step response, within each Step Task, we evaluated the responsiveness in controlling leg external force (Figure 3c). To accomplish this, we took step data from each Step Task. We segmented it into 10 individual step responses. Each segmentation consisted of a 6‐second single‐step response, 1 second before the step‐up, and 1 second after the step‐down. For each step response, we evaluated the rise time (in units of milliseconds) as the time for the signal to go from 10% to 90% of the target step size value (Kudzia et al., 2022). We calculated bandwidth (in Hz units) by dividing 0.35 by the rise time (Thompson, 2014). The bandwidth measures the maximum frequency at which a target signal can change and still be accurately tracked by the controller. Because we calculate bandwidth using rise time, bandwidth is not an independent measure of responsiveness but depends on any changes in rise time. We restricted our evaluation of the rise time and bandwidth of the response to the time interval of 0.5 s before the step target stepped up to its high value and up to 1 second after the step‐up occurred. Finally, we quantified fall time (in units of milliseconds) as the time for the signal to go from 90% to 10% of the target step size value. We evaluated the fall time between 4 and 6 s when the step signal stepped down from the target step size to 1 BW. In this paper, we use the term responsiveness to refer to rise time, bandwidth, and fall time. In robotic systems, and how we think of responsiveness in this paper, a responsive system capable of high‐fidelity tracking of a target possesses a short rise time, a wide bandwidth, and a short fall time (Azocar et al., 2018).

For each step response, within each Step Task, we evaluated the accuracy of controlling leg external force (Figure 3c). We quantified overshoot by taking the peak value reached by the signal, subtracting the value of the target step, dividing this by the size of the step, and multiplying by 100. We expressed overshoot as the percentage that the signal exceeds the target step size value. A large overshoot can indicate under‐damping in the controller—it can take longer for the system to settle into a steady state, but it may be faster to reach its target step size (Kudzia et al., 2022). We restricted our evaluation of the overshoot of the response to the time interval of 0.5 s before the step target stepped up and 1 second after the step‐up occurred. Next, we quantified steady‐state error by calculating the mean value of the signal between 2 and 4 s, subtracting the value of the target step size, and taking the absolute value. We divided this by the value of the target step size and multiplied it by 100 to express the steady‐state error as a percentage of the target value. The steady‐state error informs us how much error the control system has once it has reached the target size value and settled into its new state. A large error can indicate poor control as the system cannot match the target value well. We quantified steady‐state variability by calculating the standard deviation of the signal between 2 and 4 s, dividing this by the steady‐state mean, and multiplying this value by 100. The steady‐state variability, expressed as a percentage of the steady‐state mean, informs us how variable the system is once it has reached and settled on its new state. From pilot studies, we found that using the 2–4 second interval to measure steady‐state error and steady‐state variability was a sound assumption, as participants had reached a steady state at this point. In this paper, we use the term accuracy to refer to overshoot, steady‐state error, and steady‐state variability—an accurate system has small overshoot, small steady‐state error, and small steady‐state variability. We collectively refer to the rise time, bandwidth, overshoot, steady‐state error, steady‐state variability, and fall time as the step response characteristics.

For each Pulse Task, we evaluated the responsiveness of the response (Figure 3d). To accomplish this, we took the data from each Pulse Task. We segmented it into 10 individual step responses such that each segmentation consisted of a three‐second single‐step response, with 1.5 s before the step‐up and 1.5 s after the step‐down. Following the same definitions of responsiveness as for the Step Task, we quantified the rise time, bandwidth, and fall time for each step in every Pulse Task.

We analyzed the EMG data during the Hold Task to quantify changes in muscle frequency, one objective measure of fatigue. We first bandpass filtered the raw EMG signals from 30 to 400 Hz using a fourth‐order zero‐lag Butterworth filter, and bandstop filtered the signal from 59 to 61 Hz to remove any residual 60 Hz noise. We then determined the EMG median frequencies, a common measure of local muscle fatigue during the progression of the Hold Task (Phinyomark et al., 2012). We did this analysis every 2 s of the signal. We transformed the signal from the time domain into the frequency domain by using a Fast‐Fourier analysis (Nagata et al., 1990; Potvin & Bent, 1997). A shift to lower median frequencies over time is viewed as evidence of local muscle fatigue (Nagata et al., 1990; Stulen & DeLuca, 1981). We compared the value of the signal at the start of the first condition during the Hold task to the value of the signal at the end of each final hold task for each fatigue condition.

We used exclusion criteria for the Step Tasks and Pulse Task to remove responses that may poorly describe the measured response. For the Step Task, we used the following exclusion criteria: (Kudzia et al., 2022) the response steps up 0.5 s before the target step function steps up, (Nocella et al., 2011) the response does not step‐up within 0.5 s after the target step function steps up, (Boccia et al., 2017) the response is already greater than 10% of the target step size value during the 0.5 s leading up to the step‐up, and (Jones, 2010) the response steps down 1 s or more before the target step function steps down. For the Pulse Task, we used the following criteria: (Kudzia et al., 2022) the response rises up 0.25 s before the pulse target steps up, and (Nocella et al., 2011) the response does not fall down until greater than 0.5 s after the pulse target steps down. We removed 11 step responses that meet these exclusion criteria from all subsequent analyses.

### 3.1 Statistical analysis

To determine the effects of our protocol on fatigue, we evaluated several objective measures. The first measure was changes in mean force magnitude during the MVC task. A decrease in maximum vertical force is one indicator of fatigue (Enoka & Duchateau, 2008). We compared the mean force values from each participant's final MVC tasks completed in each condition. Our second measure was force variability during each consecutive Hold Task in each fatigue condition. Increased force variability as fatigue progresses indicates local muscle fatigue (Salomoni & Graven‐Nielsen, 2012; Singh et al., 2010). We compared the mean force variability for the final 10 s of each Hold Task repeated in each fatigue condition for each participant. To estimate baseline force variability, we calculated the mean force variability for the final 10 s of a practice Hold Task during the Pre‐Fatigue condition. Our third measure was reductions in median EMG frequency. We compared the median EMG frequencies between the first 10 s of the initial Hold Task (baseline) and the final 10 s of the last Hold Task in each fatigue condition.

For all conditions, we calculated the mean value of each step response characteristic from the first five Step Task responses for each participant immediately following the final MVC task. We performed a one‐way repeated‐measures ANOVA to determine differences in mean values across the five conditions (Training, Pre‐Fatigue, Fatigue‐1, Fatigue‐2, Fatigue‐3). Following significant main effects, we conducted post‐hoc pairwise comparisons between conditions with Bonferroni correction for multiple comparisons. We calculated Cohen's *d* to quantify effect sizes, defined as the difference between two means divided by the pooled standard deviation. Effect sizes were categorized as small (*d* ≈ 0.2), medium (*d* ≈ 0.5), and large (*d* > 0.8) according to conventional thresholds (Lakens, 2013). We used MATLAB's statistical analysis toolbox and accepted *p* < 0.05 as statistically significant. Unless otherwise stated, we present all data as mean ± standard deviation.

## 4 RESULTS

### 4.1 Participants fatigued during the protocol

**FIGURE 4:**
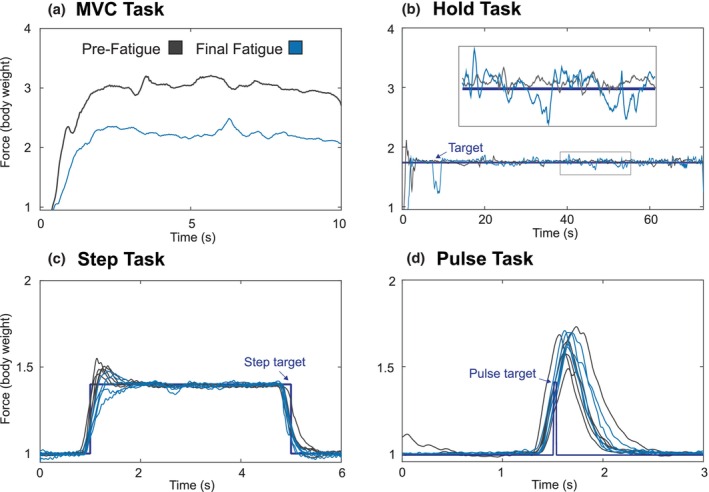
Representative participant data comparing Pre‐Fatigue (gray) and Final Fatigue (blue) conditions. (a) MVC Task showing reduced maximum force with fatigue. (b) Hold Task demonstrating increased force variability with fatigue. (c) Step Task and (d) Pulse Task show the first five responses immediately following the final MVC task in each condition, illustrating fatigue effects on force control responsiveness.

**FIGURE 5:**
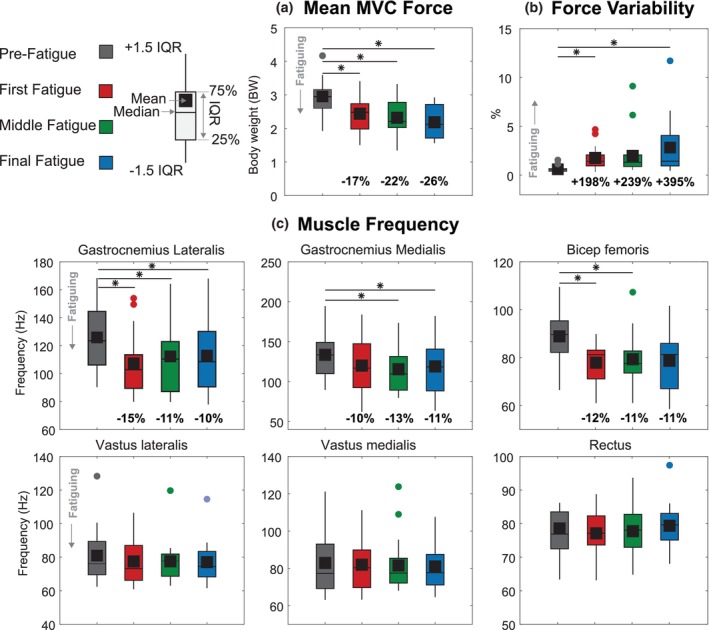
Objective measures of fatigue across experimental conditions. (a) Mean MVC vertical force decreased progressively from Pre‐Fatigue to Fatigue‐3. (b) Force variability during hold tasks increased with fatigue progression. (c) Median EMG frequency decreased in the gastrocnemius lateralis, gastrocnemius medialis, and biceps femoris muscles. Asterisks indicate significant pairwise comparisons (*p* < 0.05). Box plots show median, quartiles, and range.

Objective measures of fatigue demonstrated that participants were fatigued during the protocol. Task data for a representative participant is illustrated in Figure 4. As participants progressed through the protocol conditions, they exhibited significant reductions in mean force magnitude during the MVC Tasks (*p* = 1.30e‐12; Figure 5a). During Pre‐Fatigue, participants exerted a mean vertical leg force of 2.95 ± 0.58 BW. By the Final Fatigue condition, this decreased to 2.20 ± 0.47 BW, representing a 26.1 ± 12.3% reduction (*p* = 4.70e‐6, *d* = 1.43, large effect). Force variability during the Hold Tasks significantly increased across fatigue conditions (*p* = 2.00e‐3; Figure 5b). Pre‐Fatigue force variability was 0.61 ± 0.31%, which increased to 2.83 ± 2.93% by the Final Fatigue condition—a 4.6‐fold increase (*p* = 2.70e‐2, *d* = 1.05, large effect). EMG median frequency decreased in several lower‐leg muscles (Figure 5c). The gastrocnemius lateralis showed a 10.3 ± 8.9% reduction from 126 ± 24 Hz to 113 ± 27 Hz (*p* = 1.60e‐2, *d* = 0.51, medium effect). The gastrocnemius medialis decreased 10.9 ± 15.0% from 133 ± 28 Hz to 119 ± 34 Hz (*p* = 4.80e‐2, *d* = 0.47, small‐to‐medium effect). The biceps femoris decreased 11.4 ± 14.9% from 90 ± 12 Hz to 79 ± 13 Hz (*p* = 7.40e‐2, *d* = 0.82, large effect). The vastus lateralis, vastus medialis, and rectus femoris showed no significant changes in median frequency.

### 4.2 Fatigue led to reductions in leg force control responsiveness

**FIGURE 6:**
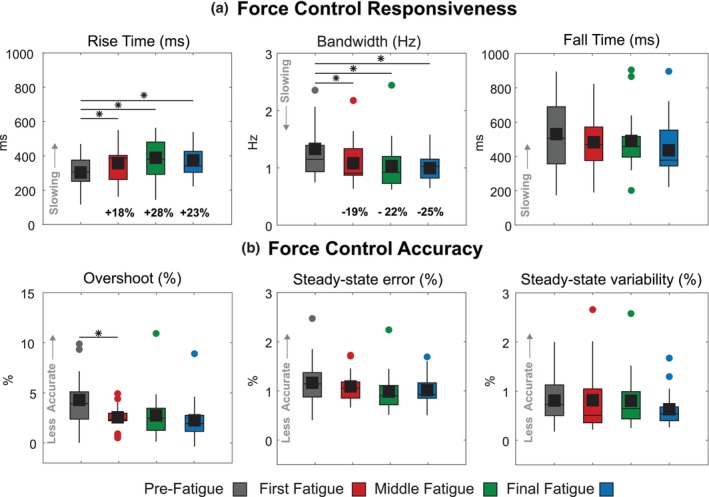
Effects of fatigue on leg force control, responsiveness, and accuracy. (a) Responsiveness measures showed significant fatigue‐related increases in rise time and decreases in bandwidth, but no change in fall time. (b) Accuracy measures remained largely unchanged, except for a transient decrease in overshoot during early fatigue that did not persist. Asterisks indicate significant pairwise comparisons (*p* < 0.05).

Fatigued participants demonstrated decreased responsiveness, with significant changes in rise time (*p* = 1.10e‐3) and bandwidth (*p* = 1.14e‐3), but not fall time (*p* = 1.09e‐1; Figure 6a). Rise time increased from 303 ± 100 ms (Pre‐Fatigue) to 373 ± 94 ms (Final Fatigue), representing a 23.3 ± 41.0% increase (*p* = 6.00e‐4, *d* = 0.72, medium‐to‐large effect). Bandwidth decreased from 1.33 ± 0.60 Hz to 1.00 ± 0.25 Hz, a 25.2 ± 17.5% reduction (*p* = 4.83e‐2, *d* = 0.72, medium‐to‐large effect). Fall time showed a trend toward reduction (531 ± 199 ms to 436 ± 174 ms, *d* = 0.51, medium effect) but did not reach statistical significance.

Force control accuracy remained largely unchanged (Figure 6b). Among accuracy metrics, only overshoot showed a transient change, decreasing from 4.30 ± 2.62% (Pre‐Fatigue) to 2.56 ± 1.19% in Fatigue‐1 (*p* = 4.70e‐2, *d* = 0.87, large effect). This reduction did not persist through subsequent fatigue conditions. Steady‐state error and steady‐state variability showed no significant changes across conditions.

### 4.3 The pulse task was resilient to the effects of fatigue

**FIGURE 7:**
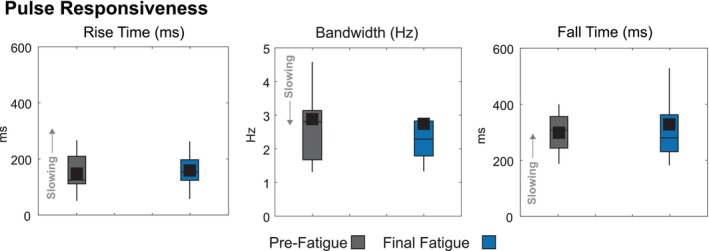
Pulse Task responsiveness, assessed by rise time, bandwidth, and fall time, comparing the Pre‐Fatigue condition to the Final Fatigue condition, Fatigue‐3. We did not evaluate the accuracy of the Pulse Task, as its primary objective was responsiveness. When the need for accuracy was removed from the objective, participants exhibited greater responsiveness than in the Step Task, which required both responsiveness and accuracy.

Fatigued participants showed no changes in responsiveness during the Pulse Task (Figure 7). Rise time remained stable from 148 ± 64 ms (Pre‐Fatigue) to 159 ± 66 ms (Post‐Fatigue), representing a 7.4% increase (*p* = 9.72e‐1, *d* = −0.17, small effect). Bandwidth showed minimal change from 2.88 ± 1.47 Hz to 2.74 ± 1.55 Hz, a 4.9% reduction (*p* = 9.15e‐1, *d* = 0.09, negligible effect). Fall time increased from 299 ± 68 ms to 328 ± 134 ms, a 9.7% increase (*p* = 7.83e‐1, *d* = −0.27, small effect). As the primary objective of the Pulse Task was responsiveness, we did not evaluate accuracy.

### 4.4 Participants exhibited higher responsiveness in the pulse task compared to the step task

Participants demonstrated greater responsiveness in the Pulse Task than in the Step Task across all measures. Rise time in the Pulse Task (148 ± 64 ms) was 155 ms faster than the Step Task (303 ± 100 ms), representing a 51.1 ± 18.9% reduction (*p* = 6.80e‐6, *d* = 1.80, large effect). Bandwidth in the Pulse Task (2.88 ± 1.47 Hz) was 1.55 Hz wider than the Step Task (1.33 ± 0.61 Hz), a 116.5 ± 87.0% increase (*p* = 4.00e‐4, *d* = 1.43, large effect). Fall time in the Pulse Task (299 ± 68 ms) was 232 ms faster than the Step Task (531 ± 199 ms), showing a 43.7 ± 34.2% reduction (*p* = 6.00e‐4, *d* = 1.50, large effect). These findings indicate that when accuracy constraints are removed, participants can generate substantially more rapid force modulation.

## 5 DISCUSSION

In this study, we investigated the impact of fatigue on the control of the vertical component of external leg forces, revealing a selective impairment where fatigue compromised responsiveness by ~25% while preserving force control accuracy. We confirmed the effectiveness of our fatigue protocol through multiple objective indicators: reductions in MVC force, increased force variability during the Hold Task, and decreased EMG median frequency across several lower‐leg muscles. These physiological changes suggest that participants experienced a diminished capacity to generate and regulate force, particularly in tasks requiring rapid adjustments. These findings align with previous research highlighting the influence of fatigue on motor control, where neuromuscular fatigue disrupts force steadiness and the ability to respond swiftly to environmental demands (Padua et al., 2005). The 4.6‐fold increase in force variability further supports this, indicating less consistent motor output under fatigue, a characteristic commonly observed in fatigued states (Jones, 2010).

Consistent with our hypothesis, fatigued participants demonstrated a marked decline in responsiveness during the Step Task, with a 23 ± 41% increase in rise time and a 25 ± 18% reduction in bandwidth, reflecting slower force modulation and potential delays in reacting to external changes. However, contrary to expectations, force control accuracy remained largely intact, except for a transient decrease in overshoot early in the fatigue phase. This suggests that participants may have used compensatory strategies to maintain precision despite reduced responsiveness. Prior studies have reported similar findings, where accuracy appears less sensitive to fatigue‐related declines, as individuals may prioritize precision over speed when controlling force output (Boccia et al., 2018). The temporary reduction in overshoot may represent an initial adaptive phase, during which participants adjusted their control strategies before deeper fatigue took hold (Branscheidt et al., 2019).

While the Step Task showed fatigue‐related declines in responsiveness, the Pulse Task remained largely unaffected. This contrast may be attributed to the differing control mechanisms underlying each task. The Step Task relies on sustained force adjustments and feedback control, both of which are sensitive to fatigue, whereas the Pulse Task involves brief, explosive force production, likely driven by feedforward control processes that are perhaps less impacted by fatigue (Buckthorpe et al., 2014; Maeda et al., 2018). Since fatigue predominantly impairs sustained force output and fine‐tuned feedback corrections, the Pulse Task's emphasis on rapid force application may have reduced its vulnerability to fatigue‐related deficits. Across all measures, participants consistently demonstrated greater responsiveness in the Pulse Task compared to the Step Task, suggesting that the absence of accuracy constraints enabled them to prioritize rapid force production. This aligns with prior research indicating that when precision demands are minimized, individuals naturally adopt faster, more explosive force control strategies (Itaguchi & Fukuzawa, 2018). The significantly shorter rise and fall times, along with the broader bandwidth observed in the Pulse Task, further highlight that when fine force modulation is unnecessary, force can be applied more quickly and efficiently. However, it is important to note that most real‐world movements—from landing a jump to pushing off during a sprint—require both responsiveness and accuracy for successful performance. The Step Task's dual demands therefore provide a more ecologically valid representation of the force control challenges encountered during daily activities and sports, where the fatigue‐induced impairments we observed would likely impact functional performance.

There are several limitations to our study. First, mechanical compliance within participants' bodies and the testing apparatus may have influenced measured forces. To minimize this, we used stiff materials and securely fixed participants in the apparatus (Kudzia et al., 2022). Second, we evaluated force control at only one submaximal level. While previous research suggests that control aspects remain similar across submaximal forces up to 60% MVC (Boccia et al., 2018; Kudzia et al., 2022), future studies should test multiple force levels. Third, our results may not generalize to other populations, such as older adults or individuals with chronic conditions. These groups could exhibit different control characteristics even before the onset of fatigue (Tracy & Enoka, 2002). As our sample size was small, and because it was not our primary interest, we did not examine sex‐specific effects. For those researchers interested in studying potential sex differences in fatigue‐related force control changes, we recommend a larger sample size than what we used here. Fourth, our MVC protocol rapidly induced substantial fatigue (26% MVC reduction), potentially obscuring dose–response relationships. Without predetermined fatigue targets (e.g., stopping the Hold Task when participants reach 10%, 20%, or 30% MVC reduction), we cannot determine how incremental fatigue levels differentially affect responsiveness versus accuracy. Future work could design a more progressive fatigue protocol with staged MVC reduction thresholds to capture the full trajectory of force control changes. Additionally, subtle inadvertent fatigue during Pre‐Fatigue testing and non‐randomized task order may have influenced baselines, though our within‐task comparisons minimize order effects. Finally, our standardized protocol may not reflect real‐world fatigue involving both physical and cognitive demands (Enoka & Duchateau, 2016; Skala & Zemková, 2022).

Our observed reductions in leg force control responsiveness are large enough that they could negatively impact agile performance. Agile performance relies on quickly and accurately responding to environmental changes by adjusting the required forces the leg exerts against the ground. A decrease in responsiveness can increase the time needed for the system to respond accurately to these changes, leading to a decline in performance. This has been illustrated in simulation experiments using robotic legs, where reductions in responsiveness, as indicated by increases in rise time and narrowing of bandwidth, were observed when the controller time delay was increased (Ashtiani et al., 2021). This has also been shown in hardware experiments with legged robots—narrowing bandwidth and increasing controller time delay resulted in the robot failing to land properly during a drop landing task (Ashtiani et al., 2021). However, the consequences of a ~25% reduction in responsiveness on a system's or individual's agility may depend on their specific abilities and the task. For instance, the MIT Cheetah, a highly agile four‐legged robot, has a leg force responsiveness that is x100 times faster than that measured in humans—a 25% reduction in responsiveness may perhaps not meaningfully affect its movement performance (Katz et al., 2019; Wensing et al., 2017). On the other hand, humans already exhibit comparatively slow rise times and narrow bandwidths, suggesting that a 25% decrease in responsiveness is likely to impair agile performance. Such fatigue‐induced temporal changes in humans have been shown to affect agile performance—fatigued athletes exhibit prolonged drop landing contact times (Zemková & Hamar, 2009) and decreased running speed (Johnston et al., 2013).

Changes in leg force control responsiveness may also increase the risk of injury during physical activities. To compensate for decreases in leg force control responsiveness, individuals might alter their body mechanics to preserve performance. For example, fatigued athletes may land from jumps with more flexed knees or shift their load from plantar flexors to knee extensors (Cortes et al., 2012). Such fatigue‐induced changes in body mechanics can compromise stability and heighten the risk of injury by placing extra stress on specific joints or muscles that are not typically as active during certain movements (Chen & Chou, 2022; Santos et al., 2019). In men's collegiate soccer, for instance, players sustain approximately 50% more injuries in the second half of matches compared to the first half (Agel et al., 2007), illustrating the impact of fatigue on injury rates.

Fatigue appears to selectively impair feedback control while preserving feedforward control mechanisms. This distinction between the effects of fatigue on feedback and feedforward control helps us understand how the nervous system adapts to fatigue and guides intervention development (Sigrist et al., 2013). Targeted interventions can strengthen feedforward control through specific training programs (Aagaard, 2003) or provide external sensory feedback to optimize performance despite fatigue (Sigrist et al., 2013). Athletes particularly benefit from these approaches, as they must maintain peak performance even when fatigued. Our Step Task, which relied on feedback control, showed significant fatigue effects, while our Pulse Task, which depended more on feedforward control, showed no changes. This pattern suggests fatigue primarily impairs sensory feedback pathways rather than neural drive. However, our limited Pulse Task assessment prevents definitive conclusions about differential effects on these control mechanisms. The timing of our tasks also influences interpretation—we assessed the Step Task after each fatigue stage, revealing a performance plateau, but compared the Pulse Task only at the end of all the fatiguing conditions. This design prevents us from determining whether rapid force production would show similar plateau patterns with progressive fatigue, an important question for future research.

## 6 CONCLUSION

We demonstrated that neuromuscular fatigue selectively impairs force control responsiveness while preserving accuracy, revealing a previously unrecognized dissociation in motor control under fatigue. This finding—a 25% decline in response speed with maintained precision—suggests the nervous system prioritizes accuracy over speed when fatigued, with important implications for understanding movement deficits and injury risk. Our method provides a quantitative framework for evaluating interventions designed to maintain motor performance under challenging conditions.

These insights can guide targeted interventions across diverse populations. Athletes might benefit from training programs that specifically target responsiveness maintenance, military personnel could use real‐time feedback systems to compensate for slowed reactions, and older adults might employ assistive technologies that accommodate reduced response speeds while leveraging preserved accuracy. Future research should refine this approach by incorporating gradual fatigue progression, testing multiple force levels, and integrating subjective fatigue measures with objective performance metrics. Such refinements will enable a more nuanced understanding of the fatigue‐performance relationship and support the development of evidence‐based strategies to preserve motor function in both everyday activities and high‐demand scenarios.

## AUTHOR CONTRIBUTIONS

P.K. and J.M.D. conceived and designed the research; P.K. performed experiments; P.K. analyzed the data; P.K., J.M.W., S.N.R., and J.M.D. interpreted the results of the experiments; P.K. prepared the figures; P.K. and J.M.D. drafted the manuscript; P.K., J.M.W., S.N.R., and J.M.D. edited and revised the manuscript; P.K., J.M.W., S.N.R., and J.M.D. approved the final version of the manuscript.

## FUNDING INFORMATION

This work is supported by the Natural Sciences and Engineering Research Council of Canada Discovery Grant (RGPIN‐2020‐04638) to J.M.D., the NSERC PGS Doctoral Scholarship, and the SFU Graduate Scholarship to P.K.

## CONFLICT OF INTEREST STATEMENT

The authors declare no competing or financial interests.

## ETHICS STATEMENT

The Office of Research Ethics at Simon Fraser University approved the study (#2020s0059).

## Data Availability

The data that support the findings of this study are openly available at https://doi.org/10.5281/zenodo.8200773.

## References

[phy270498-bib-0001] Aagaard, P. (2003). Training‐induced changes in neural function. Exercise and Sport Sciences Reviews, 31(2), 61–67. 12715968 10.1097/00003677-200304000-00002

[phy270498-bib-0002] Adam, A. , & De Luca, C. J. (2005). Firing rates of motor units in human vastus lateralis muscle during fatiguing isometric contractions. Journal of Applied Physiology, 99(1), 268–280. 16036904 10.1152/japplphysiol.01344.2004

[phy270498-bib-0033] Agel, J. , Evans, T. A. , Dick, R. , Putukian, M. , & Marshall, S. W. (2007). Descriptive epidemiology of collegiate men's soccer injuries: National Collegiate Athletic Association Injury Surveillance System, 1988–1989 through 2002–2003. Journal of Athletic Training, 42(2), 270–277. 10.4085/1062-6050-42.2.270 17710176 PMC1941292

[phy270498-bib-0003] Ashtiani, M. S. , Aghamaleki Sarvestani, A. , & Badri‐Spröwitz, A. (2021). Hybrid parallel compliance allows robots to operate with sensorimotor delays and low control frequencies. Frontiers in Robotics and AI, 8, 645748. 34312595 10.3389/frobt.2021.645748 PMC8302765

[phy270498-bib-0004] Azim, E. , & Seki, K. (2019). Gain control in the sensorimotor system. Current Opinion in Physiology, 8, 177–187. 31403088 10.1016/j.cophys.2019.03.005 PMC6688851

[phy270498-bib-0005] Azocar, A. F. , Mooney, L. M. , Hargrove, L. J. , & Rouse, E. J. (2018). Design and characterization of an open‐source robotic leg prosthesis. In B. H. Hu , J. Kuehn , & S. Haddadin (Eds.), 2018 7th IEEE International Conference on Biomedical Robotics and Biomechatronics (Biorob) (pp. 111–118). IEEE.

[phy270498-bib-0006] Benesch, S. , Pütz, W. , Rosenbaum, D. , & Becker, H. (2000). Reliability of peroneal reaction time measurements. Clinical Biomechanics, 15(1), 21–28. 10590341 10.1016/s0268-0033(99)00026-1

[phy270498-bib-0007] Bigland‐Ritchie, B. (1984). Muscle fatigue and the influence of changing neural drive. Clinics in Chest Medicine, 5(1), 21–34. 6327146

[phy270498-bib-0008] Boccia, G. , Dardanello, D. , Brustio, P. R. , Tarperi, C. , Festa, L. , Zoppirolli, C. , Pellegrini, B. , Schena, F. , & Rainoldi, A. (2018). Neuromuscular fatigue does not impair the rate of force development in ballistic contractions of submaximal amplitudes. Frontiers in Physiology, 9, 1503. 30405448 10.3389/fphys.2018.01503 PMC6207600

[phy270498-bib-0009] Boccia, G. , Dardanello, D. , Tarperi, C. , Rosso, V. , Festa, L. , La Torre, A. , Pellegrini, B. , Schena, F. , & Rainoldi, A. (2017). Decrease of muscle fiber conduction velocity correlates with strength loss after an endurance run. Physiological Measurement, 38(2), 233–240. 28099172 10.1088/1361-6579/aa5139

[phy270498-bib-0010] Branscheidt, M. , Kassavetis, P. , Anaya, M. , Rogers, D. , Huang, H. D. , Lindquist, M. A. , & Celnik, P. (2019). Fatigue induces long‐lasting detrimental changes in motor‐skill learning. eLife, 25, e40578. 10.7554/eLife.40578 PMC6443347 30832766

[phy270498-bib-0011] Buckthorpe, M. , Pain, M. T. G. , & Folland, J. P. (2014). Central fatigue contributes to the greater reductions in explosive than maximal strength with high‐intensity fatigue. Experimental Physiology, 99(7), 964–973. 24728678 10.1113/expphysiol.2013.075614

[phy270498-bib-0012] Chen, S.‐H. , & Chou, L.‐S. (2022). Gait balance control after fatigue: Effects of age and cognitive demand. Gait & Posture, 95, 129–134. 35487020 10.1016/j.gaitpost.2022.04.020

[phy270498-bib-0013] Contessa, P. , Adam, A. , & De Luca, C. J. (2009). Motor unit control and force fluctuation during fatigue. Journal of Applied Physiology, 107(1), 235–243. 19390005 10.1152/japplphysiol.00035.2009 PMC2711782

[phy270498-bib-0014] Cortes, N. , Quammen, D. , Lucci, S. , Greska, E. , & Onate, J. (2012). A functional agility short‐term fatigue protocol changes lower extremity mechanics. Journal of Sports Sciences, 30(8), 797–805. 22424559 10.1080/02640414.2012.671528 PMC3323885

[phy270498-bib-0015] Enoka, R. M. , & Duchateau, J. (2008). Muscle fatigue: What, why and how it influences muscle function. The Journal of Physiology, 586(1), 11–23. 17702815 10.1113/jphysiol.2007.139477 PMC2375565

[phy270498-bib-0016] Enoka, R. M. , & Duchateau, J. (2016). Translating fatigue to human performance. Medicine and Science in Sports and Exercise, 48(11), 2228–2238. 27015386 10.1249/MSS.0000000000000929 PMC5035715

[phy270498-bib-0017] Franklin, G. F. (2008). Feedback control of dynamic systems (p. 928). Pearson Education.

[phy270498-bib-0018] Fukuchi, R. K. , Fukuchi, C. A. , & Duarte, M. (2017). A public dataset of running biomechanics and the effects of running speed on lower extremity kinematics and kinetics. PeerJ, 5, e3298. 28503379 10.7717/peerj.3298 PMC5426356

[phy270498-bib-0019] Hermens, H. J. , Freriks, B. , Disselhorst‐Klug, C. , & Rau, G. (2000). Development of recommendations for SEMG sensors and sensor placement procedures. Journal of Electromyography and Kinesiology, 10(5), 361–374. 11018445 10.1016/s1050-6411(00)00027-4

[phy270498-bib-0020] Delsys Trigno Wireless EMG Sensors [Internet]. [cited 2024 Mar 27]. https://www.adinstruments.com/products/trigno‐emg‐sensors

[phy270498-bib-0021] Itaguchi, Y. , & Fukuzawa, K. (2018). Influence of speed and accuracy constraints on motor learning for a trajectory‐based movement. Journal of Motor Behavior, 50(6), 653–663. 29190186 10.1080/00222895.2017.1400946

[phy270498-bib-0022] Johnston, R. B., 3rd , Howard, M. E. , Cawley, P. W. , & Losse, G. M. (1998). Effect of lower extremity muscular fatigue on motor control performance. Medicine and Science in Sports and Exercise, 30(12), 1703–1707. 9861603 10.1097/00005768-199812000-00008

[phy270498-bib-0023] Johnston, R. D. , Gabbett, T. J. , & Jenkins, D. G. (2013). Influence of an intensified competition on fatigue and match performance in junior rugby league players [Internet]. Journal of Science and Medicine in Sport, 16, 460–465. 10.1016/j.jsams.2012.10.009 23245879

[phy270498-bib-0024] Jones, D. A. (2010). Changes in the force‐velocity relationship of fatigued muscle: Implications for power production and possible causes. The Journal of Physiology, 588(Pt 16), 2977–2986. 20547674 10.1113/jphysiol.2010.190934 PMC2956939

[phy270498-bib-0025] Katz, B. , Carlo, J. D. , & Kim, S. (2019). Mini cheetah: A platform for pushing the limits of dynamic quadruped control. In H. Liu , J. Qiu , & W. Huang (Eds.), 2019 International Conference on Robotics and Automation (ICRA) (pp. 6295–6301). IEEE.

[phy270498-bib-0026] Kudzia, P. (2023). Characterizing, modeling, and predicting the external ground reaction forces of legged movement [Internet]. [cited 2024 Oct 16]. https://summit.sfu.ca/item/36636

[phy270498-bib-0027] Kudzia, P. , Robinovich, S. N. , & Donelan, J. M. (2022). Characterizing the performance of human leg external force control. Scientific Reports, 12(1), 4935. 35322065 10.1038/s41598-022-08755-3 PMC8943015

[phy270498-bib-0028] Kuo, A. D. (2002). The relative roles of feedforward and feedback in the control of rhythmic movements. Motor Control, 6(2), 129–145. 12122223 10.1123/mcj.6.2.129

[phy270498-bib-0029] Lakens, D. (2013). Calculating and reporting effect sizes to facilitate cumulative science: A practical primer for t‐tests and ANOVAs. Frontiers in Psychology, 4, 863. 24324449 10.3389/fpsyg.2013.00863 PMC3840331

[phy270498-bib-0030] Maeda, R. S. , Cluff, T. , Gribble, P. L. , & Pruszynski, J. A. (2018). Feedforward and feedback control share an internal model of the arm's dynamics. The Journal of Neuroscience, 38(49), 10505–10514. 30355628 10.1523/JNEUROSCI.1709-18.2018 PMC6596259

[phy270498-bib-0031] Mohammadi, F. , & Roozdar, A. (2010). Effects of fatigue due to contraction of evertor muscles on the ankle joint position sense in male soccer players. American Journal of Sports Medicine, 38(4), 824–828. 20139329 10.1177/0363546509354056

[phy270498-bib-0032] Nagata, S. , Arsenault, A. B. , Gagnon, D. , Smyth, G. , & Mathieu, P. A. (1990). EMG power spectrum as a measure of muscular fatigue at different levels of contraction. Medical & Biological Engineering & Computing, 28(4), 374–378. 2246938 10.1007/BF02446157

[phy270498-bib-0034] Nocella, M. , Colombini, B. , Benelli, G. , Cecchi, G. , Bagni, M. A. , & Bruton, J. (2011). Force decline during fatigue is due to both a decrease in the force per individual cross‐bridge and the number of cross‐bridges. The Journal of Physiology, 589(Pt 13), 3371–3381. 21540343 10.1113/jphysiol.2011.209874 PMC3145945

[phy270498-bib-0035] Padua, D. A. , Carcia, C. R. , Arnold, B. L. , & Granata, K. P. (2005). Fatigue, vertical leg stiffness, and stiffness control strategies in males and females. Journal of Athletic Training, 40(3), 203–210. 17043698 PMC1569557

[phy270498-bib-0036] Phinyomark, A. , Thongpanja, S. , Hu, H. , Phukpattaranont, P. , & Limsakul, C. (2012). The usefulness of mean and median frequencies in electromyography analysis. In G. R. Naik (Ed.), Computational intelligence in electromyography analysis—A perspective on current applications and future challenges (pp. 195–220). InTech.

[phy270498-bib-0037] Potvin, J. R. , & Bent, L. R. (1997). A validation of techniques using surface EMG signals from dynamic contractions to quantify muscle fatigue during repetitive tasks. Journal of Electromyography and Kinesiology, 7(2), 131–139. 20719698 10.1016/s1050-6411(96)00025-9

[phy270498-bib-0038] Ranieri, F. , & Di Lazzaro, V. (2012). The role of motor neuron drive in muscle fatigue. Neuromuscular Disorders, 22(Suppl 3), S157–S161. 23182631 10.1016/j.nmd.2012.10.006

[phy270498-bib-0039] Salomoni, S. E. , & Graven‐Nielsen, T. (2012). Muscle fatigue increases the amplitude of fluctuations of tangential forces during isometric contractions. Human Movement Science, 31(4), 758–771. 22296775 10.1016/j.humov.2011.08.012

[phy270498-bib-0040] Santos, P. C. R. D. , Barbieri, F. A. , Zijdewind, I. , Gobbi, L. T. B. , Lamoth, C. , & Hortobágyi, T. (2019). Effects of experimentally induced fatigue on healthy older adults' gait: A systematic review. PLoS One, 14(12), e0226939. 31887182 10.1371/journal.pone.0226939 PMC6936857

[phy270498-bib-0041] Sigrist, R. , Rauter, G. , Riener, R. , & Wolf, P. (2013). Augmented visual, auditory, haptic, and multimodal feedback in motor learning: A review. Psychonomic Bulletin & Review, 20(1), 21–53. 23132605 10.3758/s13423-012-0333-8

[phy270498-bib-0042] Singh, N. B. , Arampatzis, A. , Duda, G. , Heller, M. O. , & Taylor, W. R. (2010). Effect of fatigue on force fluctuations in knee extensors in young adults. Philosophical Transactions of the Royal Society A ‐ Mathematical Physical and Engineering Sciences, 368(1920), 2783–2798. 10.1098/rsta.2010.0091 20439273

[phy270498-bib-0043] Skala, F. , & Zemková, E. (2022). Effects of acute fatigue on cognitive performance in team sport players: Does it change the way they perform? A scoping review. NATO ASI Series E Applied Sciences, 12(3), 1736.

[phy270498-bib-0044] Stulen, F. B. , & DeLuca, C. J. (1981). Frequency parameters of the myoelectric signal as a measure of muscle conduction velocity. IEEE Transactions on Biomedical Engineering, 28(7), 515–523. 7275132 10.1109/TBME.1981.324738

[phy270498-bib-0045] Taylor, J. L. , Amann, M. , Duchateau, J. , Meeusen, R. , & Rice, C. L. (2016). Neural Contributions to muscle fatigue: From the brain to the muscle and Back again. Medicine and Science in Sports and Exercise, 48(11), 2294–2306. 27003703 10.1249/MSS.0000000000000923 PMC5033663

[phy270498-bib-0046] Taylor, J. L. , & Gandevia, S. C. (2008). A comparison of central aspects of fatigue in submaximal and maximal voluntary contractions. Journal of Applied Physiology (1985), 104(2), 542–550. 10.1152/japplphysiol.01053.2007 18032577

[phy270498-bib-0047] Thompson, M. T. (2014). Review of signal processing basics. In Intuitive analog circuit design (2nd ed., pp. 15–52). Elsevier.

[phy270498-bib-0048] Tracy, B. L. , & Enoka, R. M. (2002). Older adults are less steady during submaximal isometric contractions with the knee extensor muscles. Journal of Applied Physiology, 92(3), 1004–1012. 11842033 10.1152/japplphysiol.00954.2001

[phy270498-bib-0049] Wensing, P. M. , Wang, A. , Seok, S. , Otten, D. , Lang, J. , & Kim, S. (2017). Proprioceptive actuator design in the MIT cheetah: Impact mitigation and high‐bandwidth physical interaction for dynamic legged robots. IEEE Transactions on Robotics, 33(3), 509–522.

[phy270498-bib-0050] Wilkins, J. C. , Valovich McLeod, T. C. , Perrin, D. H. , & Gansneder, B. M. (2004). Performance on the balance error scoring system decreases after fatigue. Journal of Athletic Training, 39(2), 156–161. 15173867 PMC419510

[phy270498-bib-0051] Zemková, E. , & Hamar, D. (2009). The effect of soccer match induced fatigue on neuromuscular performance. Kinesiology, 41(2), 195–202. https://hrcak.srce.hr/file/70903

